# Acceptability of veggie bread among Lebanese adults

**DOI:** 10.3389/fpubh.2025.1573593

**Published:** 2025-06-18

**Authors:** Asma Chanbour, Hussein F. Hassan, Dima Kreidieh, Salma Khazaal, Tareq Osaili, Khaled El Omari, Nada El Darra

**Affiliations:** ^1^Department of Nutrition and Dietetics, Faculty of Health Sciences, Beirut Arab University, Beirut, Lebanon; ^2^Department of Nutrition and Food Science, School of Arts and Sciences, Lebanese American University, Beirut, Lebanon; ^3^Department of Clinical Nutrition and Dietetics, College of Health Sciences, University of Sharjah, Sharjah, United Arab Emirates; ^4^Department of Nutrition and Food Technology, Faculty of Agriculture, Jordan University of Science and Technology, Irbid, Jordan; ^5^Laboratoire Microbiologie Santé et Environnement (LMSE), Faculty of Public Health, Doctoral School of Sciences and Technology, Lebanese University, Tripoli, Lebanon; ^6^Quality Control Center Laboratories (QCCLABS)—Industrial Development and Research Agro-Agri Center (IDRAC), Center at the Chamber of Commerce, Industry and Agriculture of Tripoli and North Lebanon, Tripoli, Lebanon

**Keywords:** bread, veggie, questionnaire, sensory, acceptability, preference, Lebanon

## Abstract

Bread is a staple food in Lebanon, but traditional refined wheat bread lacks essential nutrients. Incorporating vegetables as functional ingredients has gained interest in enhancing its nutritional profile. This study aimed to assess Lebanese adults' willingness to adopt veggie bread, given its novelty in Lebanon. An English-Arabic survey was administered to 750 Lebanese adults to evaluate bread consumption and their willingness to switch to veggie bread. Statistical analyses included frequency distribution, response percentages, internal consistency (Cronbach's alpha), and the Chi-square test for independence to examine associations between sociodemographic factors and willingness to switch. Four veggie breads were obtained and a sensory evaluation was conducted with 50 panelists using hedonic ranking, preference ranking, and food action/attitude rating tests. The sociodemographic profile of the 750 participants revealed a predominance of females (82.1%), with the majority being young adults aged 18–30 years (47.3%). The study found that freshness (64%), use-by-date (65.3%), and taste (60.7%) were the most important factors in bread selection, with price rated as “very important” by 28.5% of participants. The study found that 51.2% valued fiber addition in bread, 37.5% considered veggie bread healthier, and 52.8% would switch to veggie bread if it offered more nutrients. Statistical analysis revealed significant associations between sociodemographic factors and participants' willingness to switch to veggie bread. Education, occupation, income, and family size influenced perceptions of veggie bread's safety, health benefits, and appeal. Carrot bread received the highest acceptance and preference, followed by spinach and cabbage bread, while beetroot bread had the lowest acceptance. These results were statistically significant, as indicated by the Chi-square test (*p* < 0.008) for the hedonic ratings and *p* < 0.001 for the preference ranking. Out of 50 panelists, the majority expressed positive attitudes toward veggie bread, with 17 stating they would eat it at every opportunity and 12 frequently. This study highlights the growing interest in veggie bread among Lebanese adults, with significant factors such as health benefits and nutrition influencing their willingness to switch.

## 1 Introduction

Bread is a staple food consumed globally and holds a fundamental role in many diets, including Lebanon. It serves as significant source of energy and essential nutrients, contributing substantially to daily caloric intake (1, 2). In Lebanon, bread consumption is a key part of dietary habits, with Arabic pita bread being the most widely consumed type, along with variations such as whole wheat, multigrain, oat, bran, saj, and tannour bread (3, 4).

Despite its widespread consumption, traditional bread made from refined wheat flour lacks essential nutrients, as the milling process removes the bran and germ, which are rich in dietary fiber, vitamins, minerals, and bioactive compounds (2). Recently, plant-based ingredients like psyllium husk and flaxseed have been used to enhance bread's texture and nutritional content, serving the demand for more natural and nutritionally balanced products (5, 6).

Among functional ingredients, vegetables present significant potential for bread enrichment due to their high content of fiber, antioxidants, and bioactive compounds. The World Health Organization recommends a daily intake of at least 400 g of fruits and vegetables (F&V) to reduce the risk of non-communicable diseases such as cardiovascular disorders, type 2 diabetes, and certain cancers (7). However, global vegetable consumption remains below the recommended levels, and inadequate intake of F&V has been identified as one of the top ten risk factors for mortality (8, 9). In Lebanon, dietary patterns tend to favor grain-based products over vegetables (10), making it crucial to identify strategies that integrate vegetables into widely consumed foods like bread.

Incorporating vegetables into bread offers a practical solution to improve vegetable intake while aligning with consumer preferences. Additionally, veggie breads made without any wheat flour can serve individuals with dietary restrictions, such as those with celiac disease (CD), who follow a gluten-free diet often lacking in essential nutrients (11, 12). CD prevalence is estimated at 1% worldwide, and ~0.5% in Lebanon (13, 14). Unlike vegetable-enriched bread, which contains wheat flour along with vegetable ingredients, veggie bread refers to formulations made entirely from vegetables without the addition of any wheat flour. Thus, veggie bread could offer a healthy alternative for individuals with dietary restrictions, while also appealing to those who prefer more nutritious food options.

Consumer acceptance of veggie bread has shown promising results. A study conducted in the United Kingdom demonstrated that consumers rated the overall liking of veggie bread similarly to conventional bread, suggesting that fortification with vegetables does not necessarily compromise sensory appeal (15). However, consumer preferences for veggie bread formulations may vary based on cultural and regional dietary habits (5), highlighting the need for localized studies.

The primary aim of this study wasto assess Lebanese adults' willingness to adopt veggie bread as an alternative to traditional bread, as veggie bread is a new concept in Lebanon. Specifically, the study evaluated the sensory acceptability and preference rankings of four veggie bread formulations (fresh organic carrot, cabbage, spinach, and beetroot) to determine the most favorable option for potential market introduction. These formulations were sourced from Kenza's Kitchen, a local provider of fresh organic veggie breads, which already produces these specific types. These vegetables are rich in essential nutrients and bioactive compounds, including β-carotene, folate, vitamins A, C, and K, iron, antioxidants, and betalains, all of which contribute to improved vision, immune support, and overall health (16–19). Understanding consumer perceptions is essential for ensuring that nutritional enhancements do not compromise the sensory attributes that drive bread consumption. The findings of this study could provide valuable insights for food manufacturers seeking to develop healthier, more sustainable bread options tailored to local dietary preferences.

## 2 Materials and methods

### 2.1 Study tools

A survey-based questionnaire was initially developed in English and then translated into Arabic (20). The forward translation from English to Arabic was performed by the investigators and a translator familiar with the study's objectives. The translated versions were then compared to ensure consistency and accuracy. To confirm the quality of the translation, a back-translation from Arabic to English was conducted by a translator who had not seen the original questionnaire. This allowed for a comparison of both English versions to verify consistency. The test-retest procedure was conducted with 106 participants, who completed the questionnaire twice, with a 2-week interval between sessions (*t*_1_ and *t*_2_). This was done to assess the reliability, consistency, and performance of the Arabic version in comparison to the original English version. Additionally, face validity, reliability, and internal consistency of the survey-based questionnaire were evaluated.

### 2.2 Questionnaire development

The survey-based questionnaire consisted of three main sections. Section I focused on sociodemographic characteristics and included eight variables covering sex, age, marital status, education level, family size, occupation, monthly income, and type of bread consumed. These questions were presented in multiple-choice format. Section II comprised three subsections aimed at assessing different aspects of bread consumption: consumer motives for bread selection (12 questions), consumer opinions on bread (7 questions), and opinions on white Arabic pita bread compared to veggie bread (8 questions). The response items for these questions were based on a 5-point Likert scale, ranging from “1” (not important at all) to “5” (very important), presented in a multiple-response checkbox format. Section III included 14 questions designed to assess consumers' willingness to switch to veggie bread. Responses in this section were provided on a 3-point scale, with options for “yes”, “no”, or “maybe” also in a multiple-response checkbox format. Parts of the questionnaire were adapted from tools used in a previous study (21), while other sections were created specifically for this study. The process of developing, translating, and measuring the reliability, validity, and internal consistency of the questionnaire was guided by methods outlined in previous studies (22, 23).

### 2.3 Study population and sample size

The sample size for this cross-sectional study was calculated following the methodology outlined in a previous study (24). The inclusion criteria were Lebanese adults aged 18 years and older, residing in Lebanon, while the exclusion criteria excluded individuals under the age of 18 and Lebanese citizens living abroad. To estimate the sample size, it was assumed that ~50% of the Lebanese population had low knowledge of the topic, providing the maximum variability for sample size calculation. Using a 95% confidence interval and a 5% margin of error, the minimum required sample size was calculated to be 384 participants, based on the most recent population estimate of 5,773,493 (25). To enhance the reliability of the results, the sample size was increased, and a total of 750 participants were successfully recruited for the questionnaire.

### 2.4 Veggie bread sensory quality

Four types of veggie bread (carrot, spinach, cabbage, and beetroot) were sourced from Kenza Kitchen, located in Beirut, Lebanon. These bread types were made using fresh organic vegetables, with no addition of wheat flour, and vacuum-sealed for freshness. According to the labeling instructions, the carrot bread, for example, was made using the following ingredients: carrot, psyllium, flaxseed, rock salt, and paprika, based on Kenza's Kitchen recipe.

To assess their sensory quality, the bread was filled with Labneh, then served to 50 panelists, as recommended by a previous study (26). Three sensory evaluation methods were employed: hedonic ranking tests, a preference ranking test, and a food action/attitude ranking test. These tests were used to assess both the acceptability and preference of the veggie breads. Panelists received a tray containing four samples, each labeled with a unique 3-digit code. To refresh their tastebuds between tastings, panelists were provided with a glass of water (27). The samples were presented in a randomized order to prevent any bias. After tasting the samples in order from left to right, panelists were asked to select the most appropriate response for each question.

#### 2.4.1 Hedonic ranking test

The 5-point hedonic scale is widely used to measure food acceptability and consumer preferences, capturing a range from liking to disliking. This scale includes two positive and two negative categories with a neutral center. The term “hedonic” refers to willingness, as it evaluates how much panelists like or dislike the veggie bread. The term “hedonic” refers to the concept of willingness, as this scale measures likes and dislikes. Using the 5-point hedonic scale, 1 represented “extremely like”, 2 was “slightly like”, 3 indicated “neither like nor dislike”, 4 was “slightly dislike”, and 5 signified “extremely dislike”, as mentioned previously (28).

#### 2.4.2 Preference ranking test

A preference ranking test was conducted to determine the panelists' preferred type of veggie bread among the four options. They were asked to rank their preferences by assigning a “1^st^ choice” to their most preferred sample, “2^nd^ choice” to their second preference, “3^rd^ choice” to their third preference, and “4^th^ choice” to the least preferred sample.

#### 2.4.3 Food action/attitude rating test

The Food Action Rating Scale (FACT) was used to assess the panelists' attitudes toward the food samples, as outlined previously (29). The FACT scale is a successive-category rating system designed to measure food acceptance by assessing participants' likelihood to consume a food product based on their preferences and behavioral intentions. This scale ranges from “I would eat this every opportunity I had” to “I would eat this only if I were forced to”, reflecting different levels of willingness to consume the food.

### 2.5 Ethical approval

Before participating, all individuals were provided with a consent form that outlined the objectives of the survey-based questionnaire, ensuring anonymity and confidentiality of their responses. Participants were given the option to complete the questionnaire in either English or Arabic. The questionnaire was distributed via social media platforms, with no incentives offered and no direct contact with participants. The study received ethical approval from the Institutional Review Board at Beirut Arab University, under the code 2023-H-0147-HS-M-0517.

### 2.6 Statistical analysis

The statistical analyses conducted in this study included frequency distribution, response percentages, internal consistency (Cronbach's alpha), and Chi-square test. All analyses were performed using SPSS version 21 (SPSS Inc., Chicago, USA).

## 3 Results and discussion

### 3.1 Questionnaire reliability

The reliability of the questionnaire was assessed through three test-retest evaluations: the English version, the English-to-Arabic translation, and the Arabic version. Internal consistency was also measured to validate the reliability of the questionnaire.

#### 3.1.1 English-English version test-retest

The mean scores, standard deviations, *p*-values of paired *t*-test, Pearson correlation coefficients, and intraclass correlation coefficient (ICC) values for each section of the English version of the questionnaire are provided in Supplementary Table S1. A test-retest reliability assessment was conducted with 39 participants, who completed the English version of the questionnaire twice, with a 2-week interval between *t*_1_ and *t*_2_. A paired *t*-test showed no statistically significant differences (*p* > 0.05) across all sections, confirming the questionnaire's stability over time. Pearson correlation analysis demonstrated moderate to strong correlations across the subsections, with values ranging from 0.488 to 0.746, indicating a consistent response pattern between *t*_1_ and *t*_2_. The ICC further confirmed good agreement, with values ranging from 0.500 to 0.818. All ICC values were statistically significant (*p* < 0.01), reinforcing the reliability of the questionnaire. These results highlight the strong test-retest reliability of the English version of the questionnaire.

#### 3.1.2 English-Arabic test-retest (translation and back-translation)

The mean scores, standard deviations, *p*-values of paired *t*-test, Pearson correlation coefficients, and ICC values for each section of the translated English-Arabic versions of the questionnaire are presented in Supplementary Table S2. To assess the reliability of the translated questionnaire, a test-retest was conducted with 28 participants. They completed the English version at *t*_1_ and the Arabic version at *t*_2_ 2 weeks later. The paired *t*-test indicated that the mean scores for most sections showed no statistically significant differences (*p* > 0.05), suggesting strong consistency between the English and Arabic versions. Pearson correlation analysis showed strong correlations across all subsections (ranging from 0.663 to 0.913), indicating a high level of agreement between the two versions. Subsection III demonstrated the highest consistency (*r* = 0.913). Additionally, ICC values confirmed good reliability across all subsections, with all ICC values being statistically significant (*p* < 0.01) except for section III (*p* = 0.114), which showed moderate agreement. These results highlight the strong reliability of the translated questionnaire across both versions.

#### 3.1.3 Arabic-Arabic test-retest

The mean scores, standard deviations, *p*-values of paired *t*-test, Pearson correlation coefficients, and ICC values for each section of the Arabic version of the questionnaire are provided in Supplementary Table S3. A test-retest reliability assessment was conducted with 84 participants, who completed the Arabic version of the questionnaire twice, with a 2-week interval between *t*_1_ and *t*_2_. The paired *t*-test revealed no statistically significant differences across the sections (*p* > 0.05), suggesting good consistency over time. Pearson correlation analysis showed strong correlations across all subsections, with values ranging from 0.457 to 0.913. Subsection I demonstrated the highest correlation (*r* = 0.913). Additionally, the ICC confirmed good reliability, with all ICC values being statistically significant (*p* < 0.01), reinforcing the reliability of the Arabic version of the questionnaire.

### 3.2 Questionnaire internal consistency

The Cronbach's alpha values for all sections of the questionnaire ranged from 0.755 to 0.920. These values fall within the commonly accepted range of 0.70–0.95 (30, 31). A value above 0.7 is generally considered acceptable for demonstrating the reliability of a scale. As discussed, factors such as the number of test items, the inter-relatedness of items, and the dimensionality of the constructs can influence the value of alpha. For instance, a low alpha value might indicate a small number of questions, weak correlations among items, or heterogeneous factors being measured. In this study, Cronbach's alpha values suggest that the questionnaire demonstrates a strong internal consistency while avoiding redundancy, indicating its reliability for use.

### 3.3 Sociodemographic characteristics

The sociodemographic characteristics of the participants in section I of the questionnaire are summarized in Table 1. Out of the 750 participants, 616 were female (82.1%) and 134 were male (17.9%). The majority of participants were aged between 18 and 30 years (47.3%), followed by those aged 31–40 years (37.3%). Most participants were married (61.2%), with a smaller proportion being single (34.9%). In terms of education, 33.2% of participants held a bachelor's degree, and 22.7% had a master's degree. Regarding occupation, 23.6% were employed full-time, while 37.3% were unemployed. Household composition varied, with 53.3% of participants having 4 to 6 family members. When it comes to monthly income, 25.9% of participants earned between 1.5 and 4 million LBP, and 22.9% earned more than 6 million LBP. Finally, the majority of participants (63.1%) consumed white bread, followed by oat bread (18.3%), and a small percentage (0.4%) consumed veggie bread.

**Table 1:** Demographic characteristics of the participants (*n* = 750) from section I of the questionnaire.

**Variable**	**Frequency**	**Percent (%)**	**Valid percent (%)**	**Cumulative percent (%)**
**Gender**				
Female	616	82.1	82.1	82.1
Male	134	17.9	17.9	100
**Age group**				
18–30 years	355	47.3	47.3	47.3
31–40 years	280	37.3	37.3	84.7
41–50 years	84	11.2	11.2	95.9
51 years or older	31	4.1	4.1	100
**Marital status**				
Single	262	34.9	34.9	34.9
Married	459	61.2	61.2	96.1
Divorced	20	2.7	2.7	98.8
Widowed	9	1.2	1.2	100
**Education level**				
Basic (below high school)	132	17.6	17.6	17.6
High/technical school	184	24.5	24.5	42.1
Bachelor's degree	249	33.2	33.2	75.3
Master's degree	170	22.7	22.7	98
PhD	15	2	2	100
**Family members**				
1–3	218	29.1	29.1	29.1
4–6	400	53.3	53.3	82.4
7–9	107	14.3	14.3	96.7
More than 9	25	3.3	3.3	100
**Occupation**				
Student	92	12.3	12.3	12.3
Part-time employed	86	11.5	11.5	23.7
Full time employed	177	23.6	23.6	47.3
Business owner	95	12.7	12.7	60
Retired	20	2.7	2.7	62.7
Unemployed	280	37.3	37.3	100
**Monthly income (USD rate** = **1,500 L.B.P)**				
< 1 million LBP	98	13.1	13.1	13.1
1.5–4 million LBP	194	25.9	25.9	38.9
4.5–6 million LBP	134	17.9	17.9	56.8
More than 6 million LBP	172	22.9	22.9	79.7
I don't have an income	152	20.3	20.3	100
**Type of bread consumed**				
White bread	473	63.1	63.1	63.1
Brown bread	81	10.8	10.8	73.9
Whole wheat bread	37	4.9	4.9	78.8
Oat bread	137	18.3	18.3	97.1
Veggie bread	3	0.4	0.4	97.5
Barley bread	9	1.2	1.2	98.7
Other	10	1.3	1.3	100

### 3.4 Participants responses to subsection I (importance of bread selection)

**Figure 1:**
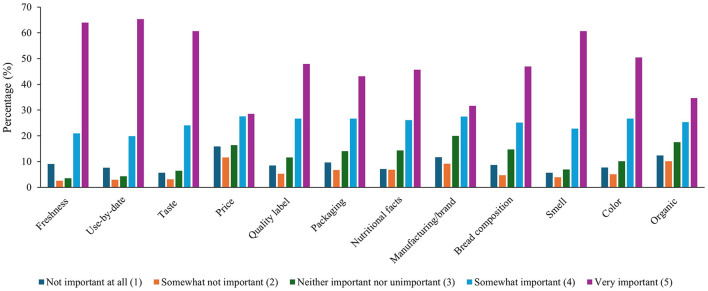
Percentages (%) of responses to subsection I (importance of bread selection) of section II.

The results for subsection I (importance of bread selection) of section II are presented in Figure 1. The aim of this section was to assess the factors influencing consumers' bread selection. The data revealed that participants considered certain factors more important than others when selecting bread. Freshness and use-by-date were identified as the most important attributes, with 64 and 65.3% of participants rating them as “very important”, respectively. Taste followed closely, with 60.7% viewing it “very important”. Price was rated as “very important” by 28.5% of respondents, but a larger proportion, 43.1%, rated it “somewhat important”. Quality label and packaging also showed importance, with 47.9 and 43.1% of participants, respectively, rating them as “very important”. Nutritional facts were also considered “very important” by 45.7%. The manufacturing brand and bread composition were rated “very important” by 31.6 and 46.9% of participants, respectively. In terms of sensory qualities, smell and color were both highly regarded, with 60.7 and 50.4% rating them as “very important”. Organic labeling, while still important, had a lower response, with 34.7% rating it as “very important”. These results indicate that freshness, use-by-date, taste, and smell are key factors influencing bread selection, while quality label, packaging, nutritional facts, and bread composition also play important roles. The findings of this study are consistent with previous research, such as the study by Sajdakowska et al., which identified sensory, nutritional, and marketing factors as key determinants in bread selection (21). However, the relative importance of these factors may vary across consumer groups, as seen in the four groups identified by Sajdakowska et al. Group 1 emphasized sensory, nutritional, and marketing motives, while Group 2 found marketing motives less influential. Group 3 focused on practical factors, and Group 4 considered practical, sensory, and nutritional aspects but rated them as less important. Overall, these results highlight the complex nature of bread consumption decisions. This study reinforces the idea that consumers weigh multiple considerations when selecting bread, with a strong preference for freshness, taste, and smell.

### 3.5 Participants responses to subsections II (consumer's opinion regarding bread) and III (opinions on white bread compared to veggie bread) of section II and section III (willingness of people to switch to veggie bread)

The responses for subsections II (Consumer's Opinion Regarding Bread) and III (Opinions on White Bread Compared to Veggie Bread) of section II, as well as section III (Willingness to Switch to Veggie Bread), are presented in Table 2.

**Table 2:** Frequency and percentages (%) of responses to subsections II (consumer's opinion regarding bread) and III (opinions on white bread compared to veggie bread) of section II and section III (willingness of people to switch to veggie bread) (*n* = 750).

**Survey statements**	**Frequency (percentage)**				
	**Not important at all (1)**	**Somewhat not important (2)**	**Neither important nor unimportant (3)**	**Somewhat important (4)**	**Very important (5)**
**Subsection II in section II: Consumers' opinion regarding bread**					
To improve the health-promoting benefits, fiber can be added to the bread	46 (6.1%)	17 (2.3%)	88 (11.7%)	215 (28.7%)	384 (51.2%)
I buy more expensive bread because I think that the price goes along with the quality	249 (33.2%)	126 (16.8%)	161 (21.5%)	126 (16.8%)	88 (11.7%)
The taste of bread is more important to me than its health-promoting benefits	143 (19.1%)	107 (14.3%)	160 (21.3%)	178 (23.7%)	162 (21.6%)
In my opinion, packaging in the bread industry is essential to ensure safety	53 (7.1%)	34 (4.5%)	90 (12.0%)	194 (25.9%)	379 (50.5%)
Information on product packaging is very important to me	75 (10.0%)	59 (7.9%)	121 (16.1%)	198 (26.4%)	297 (39.6%)
I compare information on product labels before I decide which product to choose	106 (14.1%)	84 (11.2%)	140 (18.7%)	185 (24.7%)	235 (31.3%)
I compare labels to choose products with the highest nutritional value	109 (14.5%)	73 (9.7%)	164 (21.9%)	180 (24.0%)	224 (29.9%)
**Subsection III in section II: opinions on white bread compared to veggie bread**					
Veggie bread is healthier than white bread	84 (11.2%)	69 (9.2%)	121 (16.1%)	195 (26.0%)	281 (37.5%)
Veggie bread is more expensive than white bread	106 (14.1%)	89 (11.9%)	169 (22.5%)	199 (26.5%)	187 (24.9%)
Veggie bread has higher nutrient content (especially fiber content) than white bread	67 (8.9%)	55 (7.3%)	97 (12.9%)	189 (25.2%)	342 (45.6%)
Veggie bread is less calorific than white bread	67 (8.9%)	53 (7.1%)	106 (14.1%)	179 (23.9%)	345 (46.0%)
Veggie bread is more difficult to find in shops than white bread	76 (10.1%)	54 (7.2%)	109 (14.5%)	173 (23.1%)	338 (45.1%)
Veggie bread has a better taste than white bread	109 (14.5%)	105 (14.0%)	195 (26.0%)	151 (20.1%)	190 (25.3%)
Veggie bread looks worse than white bread	142 (18.9%)	112 (14.9%)	226 (30.1%)	158 (21.1%)	112 (14.9%)
Veggie bread has a similar shelf-life to white bread	112 (14.9%)	81 (10.8%)	176 (23.5%)	172 (22.9%)	209 (27.9%)
**Survey questions**			**Frequency (percentage)**		
			**Yes**	**Maybe**	**No**
**Section III: willingness of people to switch to veggie bread**					
How often do you consume bread?			635 (84.8%)	105 (14.0%)	10 (1.3%)
How often do you purchase bread?			350 (46.7%)	305 (40.7%)	95 (12.7%)
Do you think bread made only from vegetables is safer than any other type of bread available in the market (such as oat bread, whole–wheat bread, brown bread, and white bread…) in terms of microbiological load?			174 (23.2%)	440 (58.7%)	136 (18.1%)
Do you think bread made only from vegetables contains fewer pesticide residues than any other type of bread available in the market (such as oat bread, whole–wheat bread, brown bread, and white bread…)?			157 (20.9%)	396 (52.8%)	197 (26.3%)
Do you think bread made only from vegetables is a healthier choice than any other type of bread available in the market (such as oat bread, whole–wheat bread, brown bread, or white bread…)?			284 (37.9%)	313 (41.7%)	153 (20.4%)
Do you think bread made only from vegetables is a healthier choice for patients suffering from non-communicable diseases (such as diabetes, hypertension, and heart disease…)?			393 (52.4%)	264 (35.2%)	93 (12.4%)
Do you think bread made only from vegetables is a healthier choice for people suffering from gluten sensitivity?			413 (55.1%)	229 (30.5%)	108 (14.4%)
Do you think bread made only from vegetables will have a distinct taste or texture?			422 (56.3%)	250 (33.3%)	78 (10.4%)
Do you think bread made only from vegetables would look appealing to consume?			203 (27.1%)	338 (45.1%)	209 (27.9%)
Does bread marked as “organic” make it safe to consume?			303 (40.4%)	321 (42.8%)	126 (16.8%)
Does bread marked as “organic” affect your willingness to buy it?			241 (32.1%)	297 (39.6%)	212 (28.3%)
Will you consider switching to veggie bread if you knew it is made only from vegetables, contains more fiber, vitamins, and minerals, and is less calorific than white bread?			396 (52.8%)	253 (33.7%)	101 (13.5%)
Will you consider switching to veggie bread if you knew it has the same price as white bread while it contains more fibers, vitamins, and minerals and it is less calorific?			418 (55.7%)	243 (32.4%)	89 (11.9%)
What is your degree of confidence concerning the safety and quality of bread of local origin?			185 (24.7%)	250 (33.3%)	315 (42.0%)

In subsection II, a substantial portion of participants (51.2%) considered adding fiber to bread as “very important” to improve its health-promoting benefits. However, when it came to purchasing more expensive bread based on perceived quality, only 11.7% rated this as “very important”. Taste was a crucial factor for 21.6% of participants, while 50.5% believed packaging was essential for safety. Regarding product labels, 39.6% found the information provided to be “very important”, 31.3% compared product labels before purchasing, and 29.9% compared labels to choose products with the highest nutritional value. These findings suggest that while consumers value health-oriented modifications like fiber enrichment, they remain price-conscious and highly influenced by taste, packaging, and labeling. Similarly, Sajdakowska et al. reported that respondents across various groups expressed agreement with comparable statements, with the lowest agreement observed for purchasing expensive bread, as many consumers did not equate higher prices with better quality (21). In the Lebanese market, where a medium-sized Arabic pita bread costs ~$0.38 compared to veggie bread priced between $2.80 and $3.00, the price difference could limit the acceptance of healthier bread options.

In subsection III of section II, the opinions on veggie bread compared to white bread revealed that 37.5% of participants viewed veggie bread as healthier, while 24.9% believed it was more expensive than white bread. Additionally, 45.6% agreed that it had a higher nutrient content, particularly fiber. A notable 46% perceived veggie bread as lower in calories, and 45.1% found it more difficult to find in stores. In terms of taste, 25.3% felt that veggie bread tasted better, though 14.9% thought it had a less appealing appearance than white bread. Regarding shelf life, 27.9% agreed that veggie bread had a similar shelf life to white bread. These results show a generally positive perception of veggie bread compared to white bread, with participants highlighting its potential health benefits, particularly its higher nutrient content and lower caloric value. However, challenges remain, such as the perception of veggie bread being more expensive and harder to find in stores. Although limited research exists on this topic, these findings align with a previous study by Sajdakowska et al., where most respondents disagreed with the statement suggesting bread enriched with vegetables looks worse than white bread (21).

In section III, participants' willingness to switch to veggie bread was assessed across several factors. A majority (84.8%) of participants reported consuming bread regularly, and 46.7% purchased it frequently. When considering the safety and quality of veggie bread compared to other types, 23.2% believed it was microbiologically safer, while 20.9% thought it contained fewer pesticide residues. Additionally, 37.9% of participants perceived veggie bread as a healthier choice compared to other bread types, with 52.4% considering it particularly beneficial for individuals with non-communicable diseases. Furthermore, 55.1% thought it would be a better option for those with gluten sensitivity, and 56.3% believed it would have a distinct taste or texture. However, when it came to its visual appeal, only 27.1% thought it would be attractive to consume. Regarding the concept of “organic” bread, 40.4% of participants believed that bread marked as organic was safer to consume, and 32.1% indicated that organic labeling would influence their decision to buy it. A substantial proportion of participants (52.8%) indicated they would consider switching to veggie bread if it contained more fiber, vitamins, and minerals, and was less calorific than white bread. This willingness increased to 55.7% if veggie bread were priced similarly to white bread. Despite these positive attitudes, concerns remained regarding the availability of veggie bread in stores, as 45.1% found it harder to find. Additionally, a significant portion (42.0%) expressed uncertainty about the safety and quality of locally produced bread. These findings suggest that while there is significant interest in veggie bread, particularly for its health benefits, barriers such as price, availability, and appearance may influence consumer decisions. Since veggie bread is not yet available in the Lebanese market, it is crucial to focus on raising awareness about its health benefits, safety, and appeal. This effort will be essential in encouraging consumer adoption and addressing concerns before making broader market judgments.

### 3.6 Sociodemographic determinants of the importance of Lebanese adults to switch to veggie bread

The distribution of participants' responses regarding their willingness to switch to veggie bread (section III) was analyzed across various sociodemographic characteristics, with a focus on cases where statistical significance was observed. The statistical analysis of participants' responses is detailed in Supplementary Table S4.

The Chi-square tests revealed several significant associations. For example, the frequency of bread consumption was not significantly associated with any sociodemographic factors (*p* > 0.05), but the frequency of bread purchases showed a significant difference based on gender, household size, education, occupation, and income (*p* < 0.01). Regarding perceptions of veggie bread's safety, there was a significant association between education and the belief that veggie bread is microbiologically safer (*p* < 0.01). Additionally, beliefs about veggie bread being a healthier choice for individuals with non-communicable diseases were significantly associated with education (*p* < 0.05). Furthermore, perceptions of the appeal of veggie bread's appearance were significantly associated with education, occupation, and income (*p* < 0.05, *p* < 0.01, *p* < 0.05). When examining whether bread labeled as “organic” is perceived as safe to consume, a statistically significant association was found with income levels (*p* < 0.001). Statistical analysis also revealed that family size significantly influenced participants' willingness to buy organic bread and switch to veggie bread if it offered better nutrition (*p* < 0.05). Marital status was a key factor in willingness to switch when price equivalence with white bread was considered (*p* < 0.05). Confidence in the safety and quality of locally produced bread was significantly associated with education, occupation, and income (*p* < 0.001, *p* < 0.001, *p* < 0.05). The willingness to switch to veggie bread, particularly if it is priced similarly to white bread and contains more nutrients, showed varying levels of support based on sociodemographic factors, indicating that factors such as education, occupation, and income play a crucial role in shaping consumers' willingness to adopt veggie bread.

### 3.7 Sensory evaluation of veggie bread

#### 3.7.1 Hedonic scale rate and preference ranking test results

The hedonic rating scale was used to assess panelists' acceptance of four veggie breads, as shown in Table 3.

**Table 3:** Hedonic ranking test results of 50 panelists.

**Hedonic ranking scale**	**Veggie bread**				***p*-value**
	**Carrot**	**Spinach**	**Cabbage**	**Beetroot**	
Like a lot	25^a^ (50)^*^	18^a^ (36)	19^a^ (38)	6^b^ (12)	< 0.008
Like a little	11^a^ (22)	9^a^ (18)	10^a^ (20)	23^b^ (46)	
I neither like nor dislike	9^a^ (18)	11^a^ (22)	8^a^ (16)	12^a^ (24)	
Dislike a little	2^a^ (4)	6^a^ (12)	7^a^ (14)	3^a^ (6)	
Dislike a lot	3^a^ (6)	6^a^ (12)	6^a^ (12)	6^a^ (12)	

^*^Number of panelists (percentage).

^a, b^Different letters within the same row indicate that there are significant differences of panelist liking among the veggie bread samples (at 0.05 significance level).

Carrot bread received the highest acceptance among panelists, with 50% (25/50) rating it as “like a lot” and 22% (11/50) as “like a little”, while only 10% (5/50) expressed any level of dislike. Spinach bread followed, with 36% of panelists liking it a lot, though 22% remained neutral. Cabbage bread showed a similar trend, with 38% expressing strong liking, but 16% were neutral responses and 26% reporting some level of dislike. Beetroot bread received the lowest acceptance, as only 12% of panelists rated it as “like a lot”, while 24% were neutral and 18% expressed some level of dislike.

Chi-square test revealed that significantly more panelists rated the carrot, spinach, and cabbage breads as “like a lot” compared to beetroot bread (*p* < 0.008), with carrot bread receiving the highest number of “like a lot” ratings (25 panelists, 50%). There were no significant differences in the number of panelists expressing neutral or disliking preferences among the four types of veggie breads.

Table 4 presents the results of the preference ranking test, which assessed panelists' preferences among the four veggie bread samples. Carrot bread ranked first overall, achieving the highest score of 159 points, with the majority of panelists (28/50) selecting it as their first choice. Spinach bread followed in second place with 130 points, with 20 panelists ranking it as their second choice. Cabbage bread ranked third with 122 points, marked as the third choice by 22 panelists. Beetroot bread ranked the lowest (89 points), as 31 panelists ranked it as their least preferred option.

**Table 4:** Preference ranking test results of 50 panelists.

**Preference ranking test**	**Veggie bread**				**Score**	***p*-value**
	**Carrot**	**Spinach**	**Cabbage**	**Beetroot**		
1^st^ choice	28^a^ (112)^*^	8^b^ (32)	6^b^ (24)	8^b^ (32)	159	< 0.001
2^nd^ choice	10^ab^ (30)	20^c^ (60)	16^bc^ (48)	4^a^ (12)	130	
3^rd^ choice	5^a^ (10)	16^b^ (32)	22^b^ (44)	7^a^ (14)	122	
4^th^choice	7^a^ (7)	6^a^ (6)	6^a^ (6)	31^b^ (31)	89	

^*^Number of panelists (points); 1^st^ choice (4 points), 2^nd^ choice (3 points), 3^rd^ choice (2 points), 4^th^ choice (1 point).

^a, b, c^Different letters within the same row indicate that there are significant differences of panelist preferences among the veggie bread samples (at 0.05 significance level).

In concordance with the results in Table 3, the number of panelists who ranked carrot bread as their first choice was significantly higher than those who ranked other breads first (*p* < 0.001). Similarly, significantly more panelists selected beetroot bread as their fourth choice compared to the other breads.

The hedonic scale was employed in this study due to its simplicity, making it suitable for untrained panelists and allowing straightforward data collection process. Preference testing is an essential method in sensory evaluation, capturing consumers' sensory perceptions and judgments to provide insights into product appeal. In this context, the preference ranking test was used to identify which type of veggie bread was most favored, though it does not quantify the degree of preference. It is important to distinguish between liking and preference, as a strong positive reaction to a product does not necessarily make it the most preferred overall (27, 32). The hedonic rating results showed that carrot bread received the highest acceptance, with the majority of panelists indicating they “liked it a lot” or “like it a little”, followed by spinach and cabbage bread. Beetroot bread had the lowest level of liking. Notably, most panelists expressed willingness to consume all four types of veggie bread frequently if available, indicating a general acceptance of veggie bread as a concept. This aligns with the preference ranking test results, where the carrot bread was the most preferred, followed by spinach and cabbage bread, with beetroot bread ranked the lowest.

While no studies have specifically examined bread made entirely from vegetables, research on vegetable-enriched bread offers relevant insights. For instance, Hobbs et al. (15) found that consumer liking for vegetable-enriched bread varied depending on the type of vegetable used but was not significantly different from conventional bread without enrichment. Saccotelli et al. (33) reported that bread enriched with broccoli, cauliflower, artichoke, fennel, mushroom, and zucchini was generally acceptable, with fennel-enriched bread receiving the highest sensory ratings. The general acceptance of veggie bread in this study aligns with previous research on vegetable-enriched bread, highlighting the potential for such products to appeal to consumers. Further studies could explore optimizing formulations to improve sensory attributes and expand consumer acceptance.

#### 3.7.2 Food action/attitude rating scale results

The action rating test results indicated varying levels of willingness to consume veggie bread, presented in Table 5. Among the panelists, 17 expressed strong interest, stating they would eat veggie bread at every opportunity, while 12 reported they would consume it very often. Eight panelists indicated they liked veggie bread and would eat it occasionally, whereas five mentioned they would eat it if available but would not actively seek it out. A smaller number of panelists had lower acceptance, with two stating they did not like veggie bread but would eat it on occasion, another two saying they would hardly ever consume it, and four indicating they would eat it only if forced to. These results highlight a generally positive reception, with most panelists demonstrating a willingness to incorporate veggie bread into their diet.

**Table 5:** Food action rating scale results (*n* = 50).

**Action**	**Panelists' answers**
I would eat veggie bread every opportunity that I had	17
I would eat veggie bread very often	12
I like veggie bread and would eat it now and then	8
I would eat veggie bread if available but would not go out of my way	5
I don't like veggie bread but would eat it on occasion	2
I would hardly ever eat veggie bread	2
I would eat veggie bread only if forced to	4

## 4 Conclusion

This study provides valuable insights into the factors influencing bread selection and consumer perceptions of veggie bread in the Lebanese market. The findings revealed that consumers prioritized freshness (64%), use-by-date (65.3%), taste (60.7%), and smell (60.7%) when selecting bread, with additional factors such as quality label, packaging, and nutritional facts also playing significant roles in their decision-making process. There was a strong interest in veggie bread, with 52.8% of participants indicating they would consider switching if it offered more fiber, vitamins, and minerals, and was lower in calories than white bread. However, challenges such as price, availability, and visual appeal remain key barriers to adoption. The analysis revealed that sociodemographic factors such as education, occupation, income, and family size significantly influenced Lebanese adults' willingness to switch to veggie bread, with perceived safety, nutritional value, and price equivalence with white bread being key factors. The sensory evaluation found that carrot bread received the highest acceptance and preference among panelists, followed by spinach and cabbage bread, while beetroot bread was the least favored, indicating a positive overall reception of veggie bread. It is important to note that the study's demographic skew, with a higher proportion of female and younger participants, may limit the generalizability of the results. Additionally, since the veggie bread was obtained from a local supplier, there was no control over their formulation. Consequently, nutrient composition analysis and objective textural characterization were not performed. While the potential for veggie bread in the Lebanese market is promising, addressing barriers such as price and availability, as well as enhancing its visual appeal, will be essential for broader consumer adoption. Future studies should focus on optimizing veggie bread formulations to improve its sensory attributes and explore strategies to overcome consumer concerns regarding price and availability, thereby facilitating its acceptance in the market.

## Data Availability

The original contributions presented in the study are included in the article/Supplementary material, further inquiries can be directed to the corresponding author.
