# Supplementary material for: Acceptability of veggie bread among Lebanese adults
Source: Front Public Health. 2025 Jun 18;13:1573593. doi: 10.3389/fpubh.2025.1573593 (PMC12213437; doi:10.3389/fpubh.2025.1573593)
Supplement: Supplementary file 1 [file Table_1.DOCX]

**Supplementary material**

| **Table 1.** Mean and standard deviations, Paired t-test, Pearson correlation coefficients, and ICC scores (English-English, n= 39). | | | | | | | | | | | | |
| --- | --- | --- | --- | --- | --- | --- | --- | --- | --- | --- | --- | --- |
| **Scale** | **English t_1_** | **English t_2_** | **Paired t-test*** | **Pearson Correlation** | **Intra Class Correlation**** | | | | | | | |
|  |  |  |  |  | **English t₁** | | | | **English t₂** | | | |
|  |  |  |  |  | **ICC** | **95%CI** | | **Sig** | **ICC** | **95%CI** | | **Sig** |
|  | **Mean ± SD** | **Mean ± SD** | ***p-value*** |  |  | **Lower Bound** | **Upper Bound** |  |  | **Lower Bound** | **Upper Bound** |  |
| **Subsection I in section two:**  **Bread Selection Motive** | 3.873 ± 0.640 | 3.884 ± 0.613 | 0.916 | 0.488 | 0.818 | 0.720 | 0.893 | 0.000 | 0.809 | 0.706 | 0.888 | 0.000 |
| **Subsection II in section two:**  **Consumer's opinion regarding bread** | 3.500 ± 0.684 | 3.451 ± 0.607 | 0.521 | 0.746 | 0.581 | 0.340 | 0.756 | 0.000 | 0.565 | 0.315 | 0.747 | 0.000 |
| **Subsection III in section two:**  **Opinions on white bread compared to veggie bread** | 3.635 ± 0.701 | 3.602 ± 0.663 | 0.714 | 0.678 | 0.707 | 0.542 | 0.829 | 0.000 | 0.760 | 0.625 | 0.860 | 0.000 |
| **Section III: willingness of people to switch to veggie bread** | 1.803 ± 0.288 | 1.765 ± 0.258 | 0.307 | 0.670 | 0.603 | 0.391 | 0.766 | 0.000 | 0.500 | 0.232 | 0.705 | 0.001 |

*Paired t-test: statistically significant differences at *p* < 0.05

**ICC values: statistically significant at *p* < 0.01

**Table 2.** Mean and standard deviations, Paired t-test, Pearson correlation coefficients, and ICC scores (English-Arabic, n=28).

| **Scale** | **English t_1_** | **Arabic t_2_** | **Paired t-test*** | **Pearson Correlation** | **Intra Class Correlation**** | | | | | | | |
| --- | --- | --- | --- | --- | --- | --- | --- | --- | --- | --- | --- | --- |
|  |  |  |  |  | **English t₁** | | | | **Arabic t₂** | | | |
|  |  |  |  |  | **ICC** | **95%CI** | | **Sig** | **ICC** | **95%CI** | | **Sig** |
|  | **Mean ± SD** | **Mean ± SD** | ***p-value*** |  |  | **Lower Bound** | **Upper Bound** |  |  | **Lower Bound** | **Upper Bound** |  |
| **Subsection I in section II:**  **Bread Selection Motive** | 3.799 ± 0.820 | 3.941 ± 0.794 | 0.053 | 0.898 | 0.797 | 0.659 | 0.895 | 0.000 | 0.827 | 0.709 | 0.911 | 0.000 |
| **Subsection II in section II:**  **Consumer's opinion regarding bread** | 3.291 ± 0.850 | 3.508 ± 0.665 | 0.005 | 0.913 | 0.649 | 0.396 | 0.822 | 0.000 | 0.546 | 0.218 | 0.769 | 0.002 |
| **Subsection III in section II:**  **Opinions on white bread compared to veggie bread** | 3.718 ± 0.753 | 3.685 ± 0.727 | 0.784 | 0.663 | 0.633 | 0.373 | 0.812 | 0.000 | 0.740 | 0.556 | 0.867 | 0.000 |
| **Section III: Willingness of people to switch to veggie bread** | 1.667 ± 0.233 | 1.614 ± 0.238 | 0.163 | 0.669 | 0.270 | -0.218 | 0.623 | 0.114 | 0.614 | 0.355 | 0.800 | 0.000 |

*Paired t-test: statistically significant differences at *p* < 0.05

**ICC values: statistically significant at *p* < 0.01

**Table 3.** Mean and standard deviations, Paired t-test, Pearson correlation coefficients, and ICC scores (Arabic-Arabic, n=84).

| **Scale** | **Arabic t_1_** | **Arabic t_2_** | **Paired t-test*** | **Pearson Correlation** | **Intra Class Correlation**** | | | | | | | |
| --- | --- | --- | --- | --- | --- | --- | --- | --- | --- | --- | --- | --- |
|  |  |  |  |  | **Arabic t₁** | | | | **Arabic t₂** | | | |
|  | **Mean ± SD** | **Mean ± SD** | ***p-value*** |  | **ICC** | **95% CI** | | **Sig** | **ICC** | **95% CI** | | **Sig** |
|  |  |  |  |  |  | **Lower Bound** | **Upper Bound** |  |  | **Lower Bound** | **Upper Bound** |  |
| **Subsection I in section II:**  **Bread Selection Motive** | 3.894 ± 0.894 | 3.858 ± 0.890 | 0.703 | 0.608 | 0.829 | 0.759 | 0.886 | 0.000 | 0.866 | 0.812 | 0.911 | 0.000 |
| **Subsection II in section II:**  **Consumer's opinion regarding bread** | 3.331 ± 0.803 | 3.452 ± 0.851 | 0.215 | 0.498 | 0.589 | 0.412 | 0.728 | 0.000 | 0.667 | 0.523 | 0.780 | 0.000 |
| **Subsection III in section II:**  **Opinions on white bread compared to veggie bread** | 3.411 ± 0.941 | 3.396 ± 0.879 | 0.903 | 0.297 | 0.667 | 0.525 | 0.779 | 0.000 | 0.667 | 0.526 | 0.779 | 0.000 |
| **Section III: Willingness of people to switch to veggie bread** | 1.776 ± 0.291 | 1.741 ± 0.300 | 0.332 | 0.457 | 0.582 | 0.412 | 0.720 | 0.000 | 0.659 | 0.520 | 0.771 | 0.000 |

*Paired t-test: statistically significant differences at *p* < 0.05

**ICC values: statistically significant at *p* < 0.01

Table 4. Distribution of questionnaire items by categories of sociodemographic characteristics.

|  | **Gender** | | **Age** | | **Marital Status** | | **Members number** | | **Education** | | **Occupation** | | **Income** | |
| --- | --- | --- | --- | --- | --- | --- | --- | --- | --- | --- | --- | --- | --- | --- |
|  | **Chi-Square** | ***p*-value** | **Chi-Square** | ***p*-value** | **Chi-Square** | ***p*-value** | **Chi-Square** | ***p*-value** | **Chi-Square** | ***p*-value** | **Chi-Square** | ***p*-value** | **Chi-Square** | ***p*-value** |
| **Willingness of people to switch to veggie bread** | | | | | | | | | | | | | | |
| **WVB* 1: How often do you consume bread?** | **2.5** | **0.3** | **11.0** | **0.1** | **3.9** | **0.7** | **5.3** | **0.5** | **10.8** | **0.2** | **7.1** | **0.7** | **14.8** | **0.1** |
| **WVB 2: How often do you purchase bread?** | **14.3** | **0.001** | **11.8** | **0.1** | **10.0** | **0.1** | **38.3** | **0.00** | **44.0** | **0.00** | **23.2** | **0.01** | **32.0** | **0.00** |
| **WVB 3: Do you think bread made only from vegetables is safer than any other type of bread available in the market (such as oat bread, whole–wheat bread, brown bread, and white bread…) in terms of microbiological load?** | **1.3** | **0.5** | **6.4** | **0.4** | **9.9** | **0.1** | **2.3** | **0.9** | **30.2** | **0.00** | **15.4** | **0.1** | **12.7** | **0.1** |
| **WVB 4: Do you think bread made only from vegetables contains fewer pesticide residues than any other type of bread available in the market (such as oat bread, whole–wheat bread, brown bread, and white bread…)?** | **0.2** | **0.9** | **6.2** | **0.4** | **5.7** | **0.5** | **13.1** | **0.04** | **10.2** | **0.3** | **13.0** | **0.2** | **6.0** | **0.6** |
| **WVB 5: Do you think bread made only from vegetables is a healthier choice than any other type of bread available in the market (such as oat bread, whole–wheat bread, brown bread, or white bread…)?** | **3.0** | **0.2** | **7.6** | **0.3** | **2.4** | **0.9** | **10.6** | **0.1** | **11.5** | **0.2** | **10.5** | **0.4** | **6.5** | **0.6** |
| **WVB 6: Do you think bread made only from vegetables is a healthier choice for patients suffering from NCDs (such as diabetes, hypertension, or heart disease…)?** | **3.5** | **0.2** | **9.3** | **0.2** | **2.7** | **0.8** | **3.3** | **0.8** | **18.1** | **0.02** | **12.7** | **0.2** | **6.4** | **0.6** |
| **WVB 7: Do you think bread made only from vegetables is a healthier choice for people suffering from gluten sensitivity?** | **0.8** | **0.7** | **9.6** | **0.1** | **5.0** | **0.5** | **3.3** | **0.8** | **8.3** | **0.4** | **13.2** | **0.2** | **8.8** | **0.4** |
| **WVB 8: Do you think bread made only from vegetables will have a distinct taste or texture?** | **2.0** | **0.4** | **4.4** | **0.6** | **3.4** | **0.8** | **5.1** | **0.5** | **11.6** | **0.2** | **11.9** | **0.3** | **8.3** | **0.4** |
| **WVB 9: Do you think bread made only from vegetables would look appealing to consume?** | **3.3** | **0.2** | **10.4** | **0.1** | **5.6** | **0.5** | **3.9** | **0.7** | **18.5** | **0.02** | **27.0** | **0.003** | **18.3** | **0.02** |
| **WVB 10: Does bread marked as “organic” make it safe to consume?** | **0.4** | **0.8** | **6.3** | **0.4** | **4.9** | **0.6** | **12.3** | **0.1** | **6.7** | **0.6** | **15.4** | **0.1** | **498.7** | **0.00** |
| **WVB 11: Does bread marked as “organic” affect your willingness to buy it?** | **1.0** | **0.6** | **11.2** | **0.1** | **4.1** | **0.7** | **14.8** | **0.02** | **9.8** | **0.3** | **13.4** | **0.2** | **10.7** | **0.2** |
| **WVB 12: Will you consider switching to veggie bread if you knew it is made only from vegetables, contains more fibers, vitamins, and minerals, and is less calorific than white bread?** | **5.0** | **0.1** | **6.9** | **0.3** | **10.2** | **0.1** | **17.0** | **0.01** | **9.1** | **0.3** | **4.9** | **0.9** | **3.8** | **0.9** |
| **WVB 13: Will you consider switching to veggie bread if you knew it has the same price as white bread while it contains more fibers, vitamins, and minerals and it is less calorific?** | **4.4** | **0.1** | **11.3** | **0.1** | **13.6** | **0.04** | **9.6** | **0.1** | **9.7** | **0.3** | **10.5** | **0.4** | **4.8** | **0.8** |
| **WVB 14: What is your degree of confidence concerning the safety and quality of bread of local origin?** | **0.8** | **0.7** | **6.7** | **0.3** | **6.5** | **0.4** | **8.3** | **0.2** | **36.3** | **0.00** | **35.0** | **0.00** | **18.9** | **0.02** |
|  |  |  |  |  |  |  |  |  |  |  |  |  |  |  |
| *Willingness of people to switch to veggie bread |  |  |  |  |  |  |  |  |  |  |  |  |  |  |
